# Audit and feedback in cardio– and cerebrovascular setting: Toward a path of high reliability in Italian healthcare

**DOI:** 10.3389/fpubh.2022.907201

**Published:** 2022-08-11

**Authors:** Rosella Ciurleo, Maria Cristina De Cola, Nera Agabiti, Mirko Di Martino, Placido Bramanti, Francesco Corallo

**Affiliations:** ^1^IRCCS Centro Neurolesi Bonino Pulejo, Messina, Italy; ^2^Department of Epidemiology, Lazio Regional Health Service, Rome, Italy

**Keywords:** audit and feedback, care pathway, ischemic stroke, acute myocardial infarction, neurorehabilitation

## Abstract

Adopting audit and feedback (A&F) strategies could be a suitable healthcare intervention to fulfill the challenge of monitoring and improving clinical guidelines in evidence-based medicine. Indeed, A&F is used to encourage professionals to better adhere to standard guidelines to improve healthcare performance. Briefly, an audit is an inspection of professional practice in comparison to professional standards or targets whose results are subsequently communicated to professionals in a structured manner. Although A&F strategies have been adopted in several time-dependent settings, such as for acute myocardial infarction (AMI) and stroke, interest of audits in rehabilitation care is also emerging. Recently, the Italian Ministry of Health has funded a national network project called EASY-NET, whose main objective is to evaluate the effectiveness of A&F strategies to improve healthcare practice and equity in various clinical and organizational settings in seven Italian regions. Last but not the least of these regions is the Sicily, represented within the project by the IRCCS Centro Neurolesi Bonino-Pulejo of Messina as the work package 7 (WP7). The EASY-NET WP7 is focused on the effectiveness of A&F strategies in both AMI and ischemic stroke setting, from acute to rehabilitation process of care. In this study, we described the study protocol, including the study design and methodology, providing a detailed description of the new model of A&F based on telemedicine, and discussing the possible challenges of this project.

## Introduction

Diseases of the circulatory system are the leading cause of death in Europe (~37.1% of all deaths) (1), and the most common include ischemic heart diseases (heart attacks) and cerebrovascular diseases (strokes). Notably, ischemic stroke is also the first cause of permanent disability and the second cause of dementia, with incidence oscillating between 7 and 23% in the first year after the event (2).

In Italy, >100,000 hospitalizations for acute myocardial infarction (AMI), as well as for stroke (~80% are ischemic strokes), occur every year, with 1-year mortality rates in the range of 10 and 30% (3, 4), respectively. Although the most recent statistics showed a decline in mortality, as well as a reduction in disability, premature death, and early incidence, the burden of cardio- and cerebrovascular diseases is still high, and it is expected that, within the next 20 years, there will be an increase of >30% of the prevalence rates because of the aging population (2, 5).

Implementing clinical guidelines into practice is one of the most significant challenges of evidence-based medicine. Indeed, there exists a large gap between ideal and actual care provided to such patients on which the translational research is focusing (6, 7). Previous studies showed a good level of effectiveness reached through tailored interventions for quality improvement, which are often complex and heterogeneous ranging from passive intervention as the dissemination of printed educational materials (e.g., guidelines, journal articles) (8) to active interventions as outreach visits or group education (e.g., lectures, workshops or facilitated interactive group discussions) (9), and audit and feedback (A&F) strategies (10).

A&F strategies are a well-known healthcare intervention, defined by Brehaut and Eva as “a summary of clinical performance over a specific period of time (audit), and the provision of that summary (feedback) to individual practitioners, teams, or healthcare organizations” (11). The main purpose is the improvement of quality, safety, and efficiency of the care, based on a systematic review of professional performance and the monitoring of key indicators of process quality. Essentially, an *ad hoc* team examines and verifies whether a specific clinical practice has been performed in adherence to guidelines and hospital protocols (audit), backing professionals with a report showing how they perform in relation to their peers, standards, or targets. In addition, they can identify possible actions in order to change current practice and improve performance (feedback). Audits can be used to assess individual healthcare workers' performance or that of teams, departments, hospitals, or regions.

The reliability of A&F is conditioned on the availability of collected data, method of analysis, and time devoted to this activity. Indeed, the whole process would require a well-established system of event report, data collection, purposeful management of the available information, appropriate communication of the results, careful identification of the recipients, and a time schedule for monitoring and control. Moreover, cost-effectiveness likely depends on the clinical topic (12).

Over the last two decades, A&F strategies have been used for any area of healthcare, i.e., preventive, acute, chronic, and palliative care. With the aim of improving the quality of performance, reducing failure rates, and increasing safety, as well as care access, A&F strategies have been adopted also in time-dependent settings such AMI and stroke (13–16). Any step contributes to accomplish the “mindfulness” of a successful organization, acting in the manner for individuals and teams to interact and share information, and the capability to act following that understanding.

Although most studies have focused on practice areas of the emergency and acute setting, there has recently been a growing interest in audits of rehabilitation care (17). However, it remains unclear whether A&F is more effective than other quality improvement interventions, especially when combined with any of these interventions (12). Therefore, the implementation and testing of new models of A&F in the cardio- and cerebrovascular area to improve the quality of the clinical practice from acute to rehabilitation hospitalization are needed.

### The Italian research project EASY-NET

On 15 April 2019, the Italian network project EASY-NET was started (18), which was funded by the Italian Ministry of Health and whose main objective is to evaluate the effectiveness of A&F strategies in order to improve healthcare practice and equity in various clinical and organizational settings in seven Italian regions. Therefore, EASY-NET focuses on the processes of care and/or patient outcomes that are strongly correlated with processes of care, effectiveness, and safety, besides other aspects of performance, such as timeliness, efficiency, and equity. Within the general project, the IRCCS Centro Neurolesi Bonino-Pulejo of Messina is responsible for work package 7 (WP7), focused on the effectiveness of A&F strategies in both AMI and ischemic stroke settings, from acute to rehabilitation process of care.

**Figure 1:**
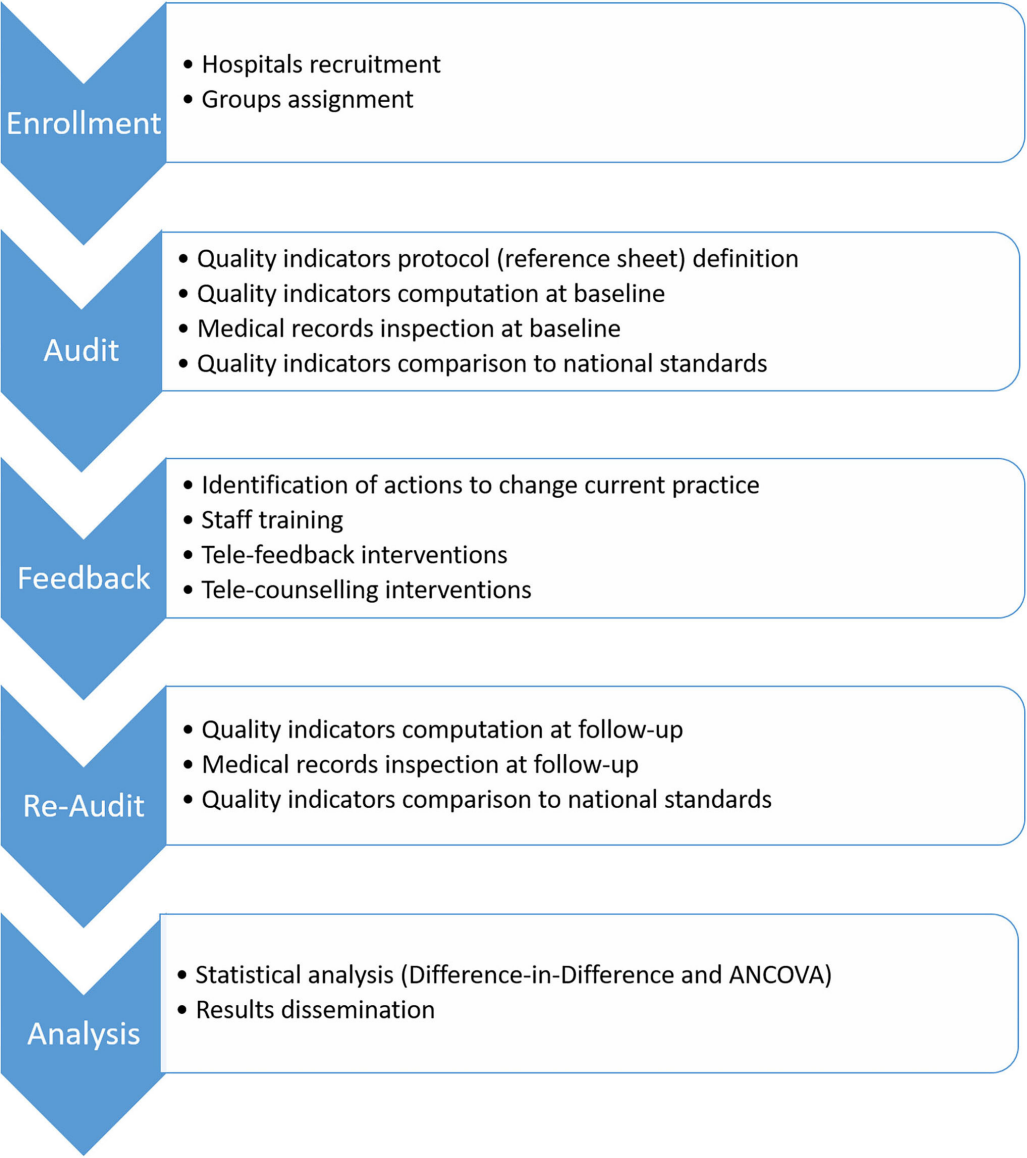
Step-by-step project procedures.

This article describes the EASY-NET WP7 study protocol, reporting the study design and methodology, and discussing expected results and challenges of this project. In addition, a detailed description of the new model of A&F based on telemedicine protocols and the intervention of an experienced psychologist is provided. The step-by-step project is reported in the flowchart depicted in Figure 1.

## Methods and analysis

### Study design

A prospective and quasi-experimental study design, since hospitals are assigned to the intervention or control group based on some characteristics, will be used to test the effectiveness of a new 1-year A&F program.

### Setting and participants

Consent was requested from the healthcare managers of the eligible Sicilian hospitals (in the areas of Messina, Catania, and Palermo) to participate in this study. The target population of the A&F intervention are healthcare workers in the context of cardio- and cerebrovascular emergency (AMI and ischemic stroke) and in neurological rehabilitation (ischemic stroke).

Eligible hospitals for the acute setting include (i) an emergency room (ii) at least a service for the treatment of cardiovascular diseases (coronary units and/or interventional cardiology services); and (iii) at least a service for the treatment of cerebrovascular diseases (stroke unit and/or interventional neuroradiology services). Eligible hospitals for the neurorehabilitative setting are provided a neurological rehabilitation unit for stroke survivors.

Enrolled hospitals have been subdivided into two groups according to geographic area and some structural characteristics such as the hospital catchment areas for AMI and stroke. The first group (group 1 or G1) includes hospitals in which the traditional A&F procedure will be performed; the second group (group 2 or G2) includes hospitals in which the new A&F model will be performed. Table 1 shows the traditional A&F procedures and the new A&F model and their differences.

**Table 1:** Traditional A&F procedures and the new A&F model and their differences.

	**Traditional A&F**	**New A&F model**	**Differences**
Audit preparation	- Establishment of a working group carrying out the audit. - Definition of reporting criteria.	- Establishment of a working group carrying out the audit. This includes the intervention of an experienced psychologist who carries out a screening on the working team dynamics in order to identify any criticalities in the relational approach. - Definition reporting criteria. The psychologist instructs the working group to adopt an effective mode of communication toward the audit recipients.	Compared with the traditional model, the new model includes the intervention of an experienced psychologist both at the stage of establishing the working group and at the stage of reporting criteria.
Clinical audit	- Definition of objectives. - Evaluation of “existing documents” by construction of appropriate checklist based on indicators produced. - Defining the strategy for data collection and analysis: methods, actions, recommendations, responsibilities, discussion, timing. - Conduct of Document Audit through document analysis: patient records, laboratories, radiology, pharmacy. - Processing data collected during audit and producing reports containing strengths and weaknesses of the process. - Sharing and communication of results (feedback phase): reports are traditionally sent *via* email.	- Definition of objectives. - Evaluation of “existing documents” by construction of appropriate checklist based on indicators produced. - Defining the strategy for data collection and analysis: methods, actions, recommendations, responsibilities, discussion, timing. - Conduct of Document Audit through analysis of documents: patient records, laboratories, radiology, pharmacy. - Processing of data collected during audit and production of reports. - Sharing and communicating the results (feedback phase): the audit team presents the results to the staff of the department undergoing the intervention, so as to discuss together the strengths and weaknesses of the process through tele-counseling sections operated with telemedicine devices positioned within the hospitals. The psychologist intervenes on the relational processes that could have induced the error (measured through error and risk indicators calculated by means questionnaires and focus groups).	Compared with the traditional model, the new model includes the intervention of an expert psychologist in the feedback phase, carried out with the support of telemedicine systems.
Implementation of improvement actions	- Definition of action plan. - Guidance and support for the change.	- Definition of action plan. During this phase, the psychologist supports the team in planning hypotheses for corrective action to be submitted to the healthcare workers, aimed at improving the organizational strategy. - Guidance and support for the change. Through the use of remote information and communication technologies typical of telemedicine, the psychologist intervenes on the communication patterns that have been able to make the care process of patients dysfunctional and implements techniques to reinforce the self-awareness and to activate the team by engaging them in precise activities.	Compared with the traditional model, the new model includes the intervention of an experienced psychologist in the phase of defining the action plan and in the phase of guiding and supporting for the change. In this latter phase the psychologist uses the telemedicine supports.
Monitoring of results	- Re-audit	- Re-audit	

### The A&F procedure: Traditional vs. new

For each setting (emergency and rehabilitation) and disease (AMI and stroke), a multidisciplinary team, including experts in the process of care in examination (e.g., risk manager, cardiologist, neurologist, physiatrist, and nursing coordinator), was constituted to conduct the A&F procedure, and a set of quality indicators (QIs) assessing hospital performance was selected and described in a specific reference sheet. Therefore, according to a checklist of information mandatory to QI computation, data were extracted and QIs computed.

Any highlighted gaps between the defined protocols and the observed management, as well as between the hospital QIs and national standards, represent the background for the planning of the hypotheses of corrective and educational interventions for healthcare professionals. On this basis, the A&F team identifies actions to change the current practice, according to the characteristics of each process of care in examination, following the corresponding guidelines and recommendations. Notably, the team sends its feedback to hospitals in the control group by email, thereby providing feedback interventions by using telemedicine to hospitals belonging to the experimental group.

The new A&F model includes a psychologist skilled in communication, who trains the A&F team to adopt an effective communication toward the recipients, reinforcing their relational techniques in the feedback phase. Thus, before the feedback, the team members undergo individual and group psychological screening through the administration of self-report tests in order to identify relational criticisms and plan effective therapeutic interventions. In addition, the psychologist, upon request, also conducts systemic therapeutic interventions aimed to correct those healthcare professionals' communication models that make the care process of the patients dysfunctional, through the use of remote information and communication technologies typical of the telemedicine.

Each center will be provided with a telemedicine platform useful for dual purposes: (i) to perform feedback as tele-counseling meetings, in which the responsible staff of the department subjected to the A&F intervention (primary and nursing coordinators) will take part, together with the A&F team and the psychologist, who will intervene on the relational processes when necessary and (ii) to perform therapeutic interventions on healthcare professionals belonging to the hospitals subjected to A&F.

#### Training and therapeutic intervention for effective communication

By administrating questionnaires and psychological tests, the psychologist identifies the communication criticalities of subjects providing the feedback and supports the team in effectively proposing the best-change strategies to healthcare professionals through the application of structured techniques ranging from the use of discussion and comparison techniques, which facilitate the processes of cognitive exchange and execution of tasks, up to problem-solving, simulation, and role-play techniques.

Therefore, after a careful examination of the problems reported by the team, the error and risk indicators will be considered through questionnaires and discussion groups. The psychologist will intervene on the relational. Timing and frequency of the meetings will be properly established, and they will involve the application of structure processes that could have led to the error. The sessions with the psychologist will include both individual and group interviews (19).

The structured techniques intervene in the group, giving it a temporary structure. The group is altered for the time provided by the technique, then returns to the original configuration, drawing the benefits gained. Among several structured techniques, the important ones are (19–23) as follows:

- Discussion and comparison techniques, which facilitate the processes of cognitive exchange, expression of the differences, creation of common codes, and performance of table tasks. Such techniques are based on the assignment of new stimuli, which can be individual, pairs, or subgroups (i.e., group divided into parts or subsets).
- Gathering techniques: “Ignite” the group meeting through self-presentation, tales of one's past, socialization, and knowledge.
- Ideation techniques: They are used to promote the creativity and invention, are carried out through brainstorming (initial phase of relaxation, chaotic phase of production of ideas, final phase of selection on the basis of rational criteria and shared), mind map (writing on the board of the free associations of the group around a concept, a network of concepts associated with each other is created), and guided imaginations (the operator speaks to the group that is in a relaxed position inducing a series of evocative images).
- Production techniques: They are used to stimulate the action and characteristics of groups with instrumental targets.
- Problem-solving techniques: They are presented a problem to be solved through the analysis of critical cases likely to be read and discussed in order to make a decision.
- Simulation techniques: You simulate “step into the shoes of,” through role-playing (role-playing, each member of the group plays a role and the focus is placed on the actor).
- Self-centered techniques: The focus of the group (point of greatest concentration of the group and the operator) is concentrated only on himself in the present moment. These techniques have an essential role in the growth of the group, which cannot be such if it lacks self-awareness.
- Directional techniques: They tend to send the group in a certain direction, minimizing the degree of freedom of the group itself. It is the operator who makes the decisions.
- Active techniques: These aim to “make people do,” to activate the group by engaging it in specific activities. These techniques are very common in educational work.
- Reflective techniques: Self-centered techniques whose main purpose is to stimulate the group to look at itself. They structure the space–time coordinates, leaving everything else free: they make the defenses of evasion or denial of reality evident.
- Diagnostic techniques: They aim to take stock, offering concrete data of the situation.
- Cognitive, emotional, instrumental techniques: The balance of the group can be tilted starting from the cognitive, emotional, or instrumental fabric (knowing, knowing how to be, and knowing how to do).

#### Psychological assessment

The protocol began with an *ad hoc* questionnaire aimed at collect information on the sociodemographic data of healthcare workers and daily life habits in relation to their employment with particular reference to their work setting. Subsequently, a battery of self-reported questionnaires was administered.

The Coping Orientations to Problem Experiences (COPE) is a 60-item self-report questionnaire that evaluates the frequency of use of 15 different coping strategies (represented by four items each) that can be adopted to manage stressful situations. In addition, they can be summarized into five factors, namely, “social support” (score 12–48), “avoidance” (score 20–80), “positive attitude” (score 12–48), “problem oriented” (score 12–48), and “religion” (score 4–16). Due to differences in the number of items that represent the five factors, mean factor scores were computed based on the number of items. Higher scores indicate that a particular coping strategy is more frequently used (24).

#### Data source

Data concerning the two acute settings will be retrospectively extracted from the inpatients' administrative database of the Sicilian Regional Health System (RHS) at two time points: in 2019 (pre-intervention) and in 2022 (post-intervention). The RHS includes different anonymous healthcare administrative databases, which can be linked with each other at patient level by a univocal key. Thus, according to the International Classification of Deceases, 9th revision, Clinical Modification (ICD-9-CM) (25), data set will include all hospital discharge records of patients older than 18 years and younger than 100 years with a primary diagnosis of AMI (ICD-9-CM codes 410.xx), or patients older than 34 years and younger than 100 years with a primary diagnosis of ischemic stroke (ICD-9-CM codes 433.x1, 434.x1, 436). Admissions lasting <2 days with a discharged home will be excluded, as well as hospitalizations of not Italian residents, transfers from another hospital with same diagnostic code, and admission preceded by discharge with diagnosis of AMI in the previous 4 weeks (ICD-9-CM codes 410.xx), or stroke in the previous 1 year (ICD-9-CM codes 430, 431, 432.x, 433.x1, 434.x1, 436). On the contrary, data in the rehabilitative setting will be extracted from the hospital administrative database, in addition to documental audits performed on inpatient medical records. Data set will include all hospital discharge records of patients older than 34 years and younger than 100 years with a secondary diagnosis of ischemic stroke (ICD-9-CM codes 433.x1, 434.x1, 436), and a primary diagnosis among the following: sequelae of cerebrovascular diseases (ICD-9-CM codes 438.xx), hemiplegia (ICD-9-CM codes 342.xx), other paralytic syndromes (ICD-9-CM codes 344.xx), dysphagia (ICD-9-CM Code 787.2), and other functional diagnoses (ICD-9-CM codes 781.0, 781.2). Admissions lasting <2 days with a discharged home will be excluded, as well as hospitalizations of not Italian residents, admissions preceded in the last 1 year by another rehabilitative hospitalization for ischemic stroke, and hospitalizations with diagnosis of hemorrhagic stroke (ICD-9-CM codes 430, 431, 432.x).

#### Quality indicators

The intervention to be effective should be supported by a monitoring process of specific indicators of changes. Thus, the audit focuses on various QIs measured in terms of structures, processes, or outcomes of care. For the EASY-NET project, a set of suitable indicators has been selected, differing for healthcare setting. Indeed, in the emergency setting only indicators whose calculation is exclusively based on the administrative RHS database will be used, whereas in the rehabilitation setting indicators whose calculation is based on *ad hoc* hospital data collection (e.g., information recorded within patients' medical records) will be used.

A total of 18 indicators (i.e., 12 for AMI and 6 for ischemic stroke) were included in the set of emergency quality indicators (EQIs) for the EASY-NET Audit, 12 of which originate from the PNE, a national program acting to support clinical and organizational audits (3). These EQIs concerned three different periods, namely, (i) hospital admission (ii) 0–30 days from the admission, and (iii) 1 year from the admission. The first 30 days can be considered as the acute management of the disease, when the patient should receive most of the guideline-based therapies and rehospitalizations may be strongly linked to the index admission; 1-year rehospitalization after the acute phase (i.e., from days 31 to 365), besides primary care it should also depend on community support provided to patients.

A total of seven indicators were included in the set of rehabilitation quality indicators (RQIs) for the EASY-NET Audit. Five out of seven RQIs were outcome indicators, such as the number of hospital admission, the average number of days for admission to inpatient rehabilitation, and the type of discharge, whereas two assess the average recovery due to rehabilitation during hospitalization.

#### Further outcomes

For the EASY-NET project, process outcomes included a post-feedback survey.

The survey collects information in three sections:

- Items on demographic about the respondents.
- Items on perceptions of the feedback report, including whether the respondents reviewed it, whether the feedback is understandable and engaging, relevant to practice, presented in a form that can be used to influence practice, and whether the process of report delivery is appropriately timed and the report is readily accessible.
- Items on intent to change behavior, including whether and how the respondents used the feedback report to change their practice.

### Statistical analysis

Descriptive statistics will be calculated for all variable targets. Continuous variables will be expressed as mean ± standard deviation, whereas categorical variables in frequencies and percentages. Student's *t*-test will be used to compare means, and the chi-squared test to compare proportions.

To assess whether the feedback intervention will lead to greater improvements in the “treatment group” vs. the “control group,” difference in differences models and multilevel models will be carried out, adjusting for multiple covariates at baseline. No interim analyses are planned. Subgroup analyses will be performed on individual hospitals.

Statistical analysis will be performed by using the open-source software R version 4.0.5. A *p* < 0.05 will be considered statistically significant.

### Study status

Data collection commenced on 15 April 2019 and will be completed on 15 August 2023. The timelines of the initial project are reported in Table 2. Indeed, the project duration is of 36 months, but it has obtained an extension for the end of the activities.

**Table 2:** Timelines of the EASY-NET WP7 project.

**Procedure task**	**Months**
Definition of the emergency quality indicators' (EQIs) and Rehabilitation quality indicators' (RQIs) protocol. Definition of the new A&F intervention	Apr 2019–Sep 2019
Hospital's recruitment and group's assignment	Oct 2019–Jan 2020
Audit: calculation of EQIs and RQIs at baseline	Feb 2020–Apr 2020
Staff training to perform the feedback intervention	May 2020
Realization of the feedback interventions	Jun 2020–May 2021
Re-Audit: calculation of EQIs and RQIs at follow-up	Jun 2021–Aug 2021
Statistical analysis	Sep 2021–Dec 2021
Results dissemination	Jan 2022–Mar 2022

## Discussion

There is evidence of a significant gap between the quality and safety of care that patients overall receive and the recommended standards.

The written definition of the responsibilities and tasks for the members of the medical team, the use of checklists, an effective communication in the medical team, and the patient feedback are important parts of the quality control and risk management in order to improve the patient safety. However, it has been seen that such actions are often missing in many clinical settings (26).

The A&F interventions are recognized as part of a strategy for improving performance and supporting quality and safety in healthcare systems.

To the best of our knowledge, this is the first project of A&F performed in Southern Italy and the first project nationwide that implemented an A&F intervention in rehabilitative settings, where effectiveness studies are limited. Indeed, it is not common practice to carry out A&F activities in Sicilian hospitals. Consequently, it was considered appropriate to choose indicators comparable with national reference targets, at least in the emergency setting. In addition, this is the first study that experiences a psychologist's intervention and the use of remote information and communication technologies typical of telemedicine.

Despite widespread use of A&F in healthcare systems as a strategy for quality improvement, uncertainties remain about its effectiveness (10). This could be related to variability of the interventions that do not always take into account human factor aspects (communication, leadership and followership, team working), which, in addition to the technical ones, are the key to building quality and reliability in healthcare. The new model of A&F requires a change in the preparation and delivery of the entire feedback process. Systematic observation and analysis of technical and non-technical skills components in the new format for clinical audit will turn an administrative review process into a highly educational experience that people are looking for.

We believe that a psychological model that supports A&F throughout its journey can improve the quality of work choices and the work environment itself. Indeed, the conceptual theory of psychosocial safety climate (PSC is based on the perspectives of occupational stress, psychosocial risk, and organizational climate literature (27). PSC is a specific component of organizational climate related to freedom from psychological harm at work (28). It reflects management's commitment to the psychological health of employees and the priority they give to safeguarding psychological health over production needs. Like organizational climate, PSC is conceptualized as a property of the organization, consisting of the aggregate perceptions of individuals within the organization regarding management's commitment to protecting their psychological health and safety (28). The PSC construct is largely derived from the idea that individuals ascribe meaning to their work environment.

In addition, many studies in the literature have highlighted how problems at work affect the development of mental health issues. Phenomena such as stress, bullying, and burnout are major contributors to a job done poorly (29). Indeed, acting on adaptive strategies, such as coping, dysfunctional phenomena that affect mental health can be prevented. Therefore, we hypothesized that even in the A&F model working on motivational and adaptive levels can significantly affect the care pathways. Therefore, more assurance could be given to healthcare workers and consequently provide better service to users/patients.

We expect that the new A&F model results in an overall improvement in the performance of healthcare providers and the quality and safety of care received by emergency and rehabilitation patients through behavior and action changes based on the development of critical thinking guided by the presence of the psychologist.

The COVID-19 pandemic determined a health emergency that impacted standard care processes. In this context, the A&F interventions may become a concrete tool able to support healthcare professionals in proactive actions aimed at reducing the risk associated with infection (30, 31). In particular, the use of ICT could lead to major facilities in the procedure of A&E, such as the ones experimented in this project.

## Limitations

The main limitation of this project is the recruitment and availability of healthcare professionals to receive the information for culture change. However, the A&F phase is carefully supervised and monitored by a central steering committee, which is responsible for identifying and training, each hospital, and the staff responsible for conducting the audits.

In addition, in the absence of benchmarks (e.g., in the rehabilitation setting), there is a difficulty in defining appropriate RQIs to detect behavior change.

## Ethics statement

As the study did not directly involve patients, data are retrospectively collected and extracted in aggregate form; this study did not require the approval of the Ethics Committee, in accordance with the current rules of our hospital. However, all target population of the A&F intervention will provide written informed consent to participate in the study. Results will be disseminated through journals and conferences, and will be particularly relevant for healthcare managers and clinicians intending to implement strategies for quality improvement based on an effective communication among different healthcare workers, as well as for researchers seeking to refine the structure and evaluate the effectiveness of such programs.

## Author contributions

NA coordinated the development of the EASY-NET project. PB and RC coordinated the development of the WP7. MDM and MCDC designed the study, designed the hospitals' stratification and the QIs protocol. PB, RC, MCDC, and FC designed the new A&F model. RC, MCDC, and FC drafted the manuscript. FC designed both the training and therapeutic intervention for effective communication. NA and PB critically revised the manuscript. All authors contributed to the article and approved the submitted version.

## Funding

This project was supported by the Italian Ministry of Health (NET-2016-02364191). The funding sources had no role in the analysis, writing, or decision to submit the manuscript.

## Conflict of interest

The authors declare that the research was conducted in the absence of any commercial or financial relationships that could be construed as a potential conflict of interest.

## Publisher's note

All claims expressed in this article are solely those of the authors and do not necessarily represent those of their affiliated organizations, or those of the publisher, the editors and the reviewers. Any product that may be evaluated in this article, or claim that may be made by its manufacturer, is not guaranteed or endorsed by the publisher.

## Abbreviations

AMI: Acute myocardial infarction
A&F: audit and feedback
WP7: work package 7
QIs: quality indicators
COPE: Coping Orientations to Problem Experiences
RHS: Regional Health System
ICD-9-CM, International Classification of Deceases, 9th revision: Clinical Modification
EQIs: emergency quality indicators
RQIs: rehabilitation quality indicators.
