# Supplementary material for: Audit and feedback in cardio– and cerebrovascular setting: Toward a path of high reliability in Italian healthcare
Source: Front Public Health. 2022 Aug 11;10:907201. doi: 10.3389/fpubh.2022.907201 (PMC9403250; doi:10.3389/fpubh.2022.907201)
Supplement: Supplementary file 1 [file Data_Sheet_1.docx]

Supplementary Material

# Emergency quality indicators (EQIs) - Acute Myocardial Infarction (AMI)

| **Name** | **Description** |
| --- | --- |
| Number of AMI admissions | Number of hospitalisations for episodes of AMI |
| Number of AMI STEMI admissions | Number of hospitalisations for episodes of AMI STEMI |
| Proportion of 90’ PTCA for AMI STEMI | Proportion of episodes of AMI STEMI treated with PTCA within 90 minutes. |
| Proportion of 48h PTCA for AMI STEMI | Proportion of episodes of AMI STEMI treated with PTCA within 48 hours. |
| Timeliness of intervention for AMI STEMI | Proportion of episodes of AMI STEMI treated with PTCA within 90 minutes out of total episodes of AMI STEMI treated with PTCA within 12 hours. |
| 30-days mortality after hospital admission | Proportion of deaths occurring within 30 days from the date of first admission to the ER for AMI. |
| 30-days mortality with 90’ PTCA | Proportion of deaths occurring within 30 days for AMI STEMI episode treated with PTCA within 90 minutes. |
| 30-days mortality with 48h PTCA | Proportion of deaths occurring within 30 days for AMI STEMI episode treated with PTCA within 48 hours. |
| 30-days mortality with PTCA over 48 hours | Proportion of deaths occurring within 30 days of AMI STEMI episode treated with PTCA over 48 hours. |
| 30-days mortality without PTCA | Proportion of deaths within 30 days of AMI episode not treated with PTCA. |
| Intra-hospital mortality | Proportion of deaths occurring during hospitalisation for AMI. |
| Time spent in the ER before transfer to a level II DEA | Average time between access to the ER of a level I DEA and discharge for transfer to a level II DEA. |

# Emergency quality indicators (EQIs) - Ischemic Stroke

| **Name** | **Description** |
| --- | --- |
| Number of ischemic stroke admissions | Number of hospitalisations for episodes of ischemic stroke. |
| 30-days mortality after hospital admission | Proportion of deaths occurring within 30 days from the date of first admission to the ER by ischemic stroke. |
| Intra-hospital mortality | Proportion of deaths occurring during hospitalisation due to ischemic stroke. |
| 30-day hospital readmissions | Proportion of hospital admissions for ischemic stroke occurred within 30 days by a discharge for ischemic stroke. |
| Proportion of MACCE at 6 months | Proportion of major cardiovascular and cerebrovascular events (MACCE) occurring within 6 months by discharge for ischemic stroke. |
| Proportion of continuity of care within 30 days | Proportion of discharged from a stroke unit followed within 30 days by an admission to a rehabilitation unit. |

# Rehabilitation quality indicators (EQIs) - Ischemic Stroke

| **Name** | **Description** |
| --- | --- |
| Number of ischemic stroke admissions in a rehabilitation unit | Number of hospitalisations in stroke rehabilitation unit. |
| Average waiting time for rehabilitation admission | Average waiting time for a stroke rehabilitation unit admission after the hospitalization in acute stroke unit. |
| Proportion of protected discharges | Proportion of protected discharges after the hospitalization in stroke rehabilitation unit. |
| Proportion of discharges at home | Proportion of discharges at home after the hospitalization in stroke rehabilitation unit. |
| Proportion of acute transfers | Proportion of transfers from rehabilitation to acute stroke unit. |
| Average recovery due to hospitalization | Average functional recovery after hospitalization in stroke rehabilitation unit. |
| Proportion of objectives achieved | Proportion of discharged where patients achieved all rehabilitation objectives. |
